# Multi-level posterior cervical-only fusion and instrumentation with versus without extension to C1: A short-term outcomes analysis using a large database

**DOI:** 10.1016/j.xnsj.2026.100897

**Published:** 2026-05-06

**Authors:** Carla Guedikian, Shahabeddin Yazdanpanah, Hana-Joy E. Hanks, Sultan Baz, Yu-Po Lee, Emily S. Mills, Nitin N. Bhatia, Don Y. Park, Sohaib Z. Hashmi, Hao-Hua Wu

**Affiliations:** a School of Medicine and Dentistry, University of Rochester, Rochester, NY 14642, United States; b College of Medicine, Northeast Ohio Medical University, Rootstown, OH 44272, United States; c Department of Orthopaedic Surgery, University of California, Orange, CA 92868, United States

**Keywords:** Multi-level, Posterior, Cervical, Subaxial, Atlantoaxial, Adverse event, Infection, NSQIP

## Abstract

**Background Context:**

Few studies have compared outcomes of posterior cervical fusions and instrumentations with versus without extension to C1. Therefore, this study evaluates such 30-day adverse events, comparing multi-level constructs extending to C1 (C1) with subaxial fusions (SF) at C2 and below, to better define complication profiles.

**Methods:**

The ACS-NSQIP database (2014–2023) was queried for patients undergoing multi-level posterior cervical-only fusions and instrumentations using CPT codes 22600 (SF) and 22595 (C1), each paired with 22842. Cases with missing key variables or involving occiput, thoracic, or lumbar arthrodesis were excluded, and coding restrictions prevented cohort overlap. The primary outcome was any adverse event (AAE), with additional subgroup analyses. Propensity score matching (PSM) of relevant covariates balanced cohorts, with standardized mean differences <0.1 considered acceptable. Statistics included chi-squares, independent t-tests, multivariable regressions with odds-ratios (ORs), and threshold analyses, when appropriate.

**Results:**

Each cohort had 281 patients after PSM: SF (mean age=50.2±10.6; BMI=28.1±6.1 kg/m^2^; 52.7% male) and C1 (age=50.7±13.0; BMI=27.8±5.6 kg/m^2^; 52.7% male). Admission-to-operation interval (0.6±1.8 vs. 1.5±2.6 days; p<.001), and operative time (157.8±76.1 vs. 193.7±74.9 minutes; p<.001) were higher in C1 patients. AAE occurred in 13.9% of SF and 18.9% of C1 patients (p=.111), with no significant differences in major (11.0% vs. 14.6%), minor (6.1% vs. 7.1%), or infection (4.3% vs. 6.1%) subgroupings. Operative time (OR=1.009; p<.001) and admission-to-operation interval (OR= 1.226; p=.016) were associated with AAE in SF patients, whereas no C1 predictors were identified. Threshold analysis identified covariate-adjusted inflection points at operative time 296 minutes (95th percentile) and admission-to-operation interval 0.98 days (81st percentile), above which AAE rates increased in SF patients (both corrected p<.01).

**Conclusions:**

SF and C1 patients demonstrated plausibly comparable short-term outcomes, suggesting that extension of cervical-only fusion and instrumentation constructs through the atlantoaxial joint does not substantially increase early postoperative risk. Attention to perioperative timing may be warranted, and larger, longer-term studies are needed for corroboration.

**Level of Evidence:**

Level III.

## Background

Multi-level posterior cervical fusion and instrumentation is a mainstay in the surgical management of cervical spine pathology, including deformity, trauma, and degenerative pathology, with widespread utilization expected to continue growing [1,2]. Surgical techniques and instrumentation continue to advance, allowing surgeons to address complex, multi-level disease through constructs extending into the upper cervical spine [2,3]. With an aging population and indications for surgery expanding, these longer constructs are becoming more common, resulting in a greater need to understand potential perioperative complications [[4], [5], [6]]. Although surgical technique has improved, posterior cervical fusion has been associated with meaningful complication rates, and whether proximal construct extent shapes that risk remains an underexplored question.

Extending the construct into the atlantoaxial region can introduce several anatomical and biomechanical variables that subaxial surgery does not [7]. The atlantoaxial complex is a primary contributor to rotational motion of the cervical spine and neighbors the vertebral arteries as well as the upper cervical nerves, making instrumentation in this area higher-risk and more technically demanding [[8], [9], [10]]. Moreover, patients undergoing constructs that extend to C1 may present with different complications and complication rates: one such example being an increased risk for dysphagia [11,12].

Prior studies have largely focused on outcomes following subaxial constructs, with comparatively limited data examining extension to C1. For instance, comparisons of constructs terminating at C2 versus C3/C4 found no meaningful difference in adverse event rates [13]. In contrast, registry data comparing occipitocervical fusion with atlantoaxial fusion demonstrated higher 30-day complication rates in the occipitocervical cohort (40.9% vs. 26.3%), hinting that anatomical region, not construct length, may influence early morbidity [14]. Whether this applies to constructs stopping at C1 versus those confined below has not been directly examined. To address this knowledge gap, this study compares 30-day adverse events between multi-level posterior cervical-only fusions and instrumentations extending to C1 versus those confined to the subaxial spine at C2 and below, aiming to better define early complication profiles and identify perioperative factors associated with increased complication risk, under the null hypothesis that no difference exists between these construct groups.

## Methods

### Data source

Data for this study was sourced from the American College of Surgeons National Surgical Quality Improvement Program (ACS-NSQIP) database. As a multi-center, United States-based, and prospectively-collected surgical registry, NSQIP utilizes standardized definitions and trained clinical reviewers to systematically document 30-day postoperative outcomes. NSQIP captures a range of variables, including patient demographics, comorbidities, intraoperative factors, and postoperative outcomes. Its 2023 reporting cycle included nearly 700 sites and over 990,000 cases, tracking over 270 distinct variables [15]. Inter-rater reliability audits have confirmed the validity of the program’s data, which have demonstrated disagreement rates of approximately 2% [16,17]. Given the absence of identifiable patient information within the dataset, this study qualified as nonhuman subjects research and thus was exempt from formal institutional review board approval.

### Patient selection

The NSQIP database was queried to identify patients who underwent multi-level posterior cervical-only arthrodesis and instrumentation between 2014 and 2023 (the latest contemporary decade) using a collection of primary current procedural terminology (CPT) codes. CPT 22600 (arthrodesis, posterior or posterolateral technique, single interspace; cervical below C2 segment) defined the subaxial cervical fusion (SF) cohort, while 22595 (arthrodesis, posterior technique, atlas-axis [C1-C2]), defined the atlantoaxial fusion (C1) cohort. To focus on multi-level posterior constructs, both cohorts were restricted to cases that also superimposed CPT 22842 (posterior segmental instrumentation; 3-6 vertebral segments).

To limit construct-length disparities that may have captured noncervical primary procedures, cases involving longer constructs were excluded: a consortium consisting of CPT codes 22843 (posterior segmental instrumentation; 7-12 vertebral segments) and 22844 (posterior segmental instrumentation; 13 or more vertebral segments). To only include cervical fusion cases-of-interest, additional superimposed CPT codes excluded from these cohorts were 22610 (arthrodesis, posterior or posterolateral technique, single interspace; thoracic), 22612 (arthrodesis, posterior or posterolateral technique, single interspace; lumbar), and 22590 (arthrodesis, posterior technique, craniocervical [occiput-C2]). To prevent potential cohort overlap and confirm true subaxial cases, CPT code 22595 (arthrodesis, posterior technique, atlas-axis [C1-C2]) was additionally excluded from the SF cohort. Cases were also excluded if demographic variables (age, sex, height, weight) or perioperative variables (American Society of Anesthesiologists [ASA] classification, functional status, or comorbidity data) were unavailable. To minimize potential bias introduced by acute physiological instability at the time of surgery, patients with recent transfusion, ventilator dependence, or preoperative sepsis were excluded as well. This set of selection criteria was informed by previously published NSQIP-based outcomes studies [[18], [19], [20]].

### Primary outcome

The primary outcome was the development of any postoperative adverse event (AAE) within 30 days of surgery. Included complications under the umbrella of AAE were as follows: surgical site infection (superficial, deep, organ/space), urinary tract infection (UTI), wound dehiscence, sepsis, pneumonia, acute kidney injury (AKI), unplanned reintubation, prolonged ventilator support (>48 hours), deep vein thrombosis (DVT), bleeding requiring transfusion, pulmonary embolism (PE), cerebrovascular accident (CVA)/stroke, myocardial infarction (MI), cardiac arrest, return to the operating room (ROR), and death. Notably, indications for ROR were categorized using associated International Classification of Diseases-10 (ICD-10) diagnosis codes, when available. The generation of the composite endpoint of AAE was adapted from prior NSQIP-based literature [18,21].

### Secondary outcomes

To further characterize composite AAE, adverse events were divided by severity. Major adverse events included death, CVA/stroke, MI, cardiac arrest, AKI, PE, DVT, unplanned reintubation, prolonged ventilator support, ROR, postoperative transfusion, deep infection, organ/space infection, and sepsis. Minor adverse events included superficial surgical site infection, wound dehiscence, UTI, and pneumonia. An additional infection-focused subgroup was created, collating all surgical site infections (superficial, deep, organ/space) with UTI and sepsis. These categorizations were tandemly adapted from prior NSQIP-based literature [22,23].

### Statistical analyses

Statistical analyses were conducted using RStudio (version 2026.01.0+392; R Foundation for Statistical Computing, Vienna, Austria), the IBM Statistical Package for the Social Sciences (SPSS; version 31.0; IBM Corp., Armonk, NY), and Microsoft Excel for Microsoft 365 Apps for Enterprise (version 2511; Microsoft Corp., Redmond, WA). For cohort balancing, 1:1 propensity score matching (PSM) of key baseline demographics and available comorbidities was performed prior to outcomes analyses, supplemented by assessment of postmatching covariate balance, and with standardized mean differences (SMDs) <0.1 considered indicative of adequate execution amidst minimal residual confounding [24]. Continuous variables are reported as means with standard deviations (SDs) and categorical/binary variables as counts (N) with corresponding percentages (%). Inter-arm comparisons were performed using Pearson’s chi-square testing for categorical variables when cell counts were adequate, with Fisher’s exact tests used for sparse categorical data. Continuous variables were compared using independent two-tailed t-tests.

Exploratory multivariable logistic regression was used to evaluate associations between covariates and the primary endpoint of AAE, with results reported as odds ratios (ORs) and 95% confidence intervals (CIs). Covariate selection was informed by prior NSQIP-based literature and emphasized demographic characteristics and common comorbid conditions available within the database. Additionally, screening was performed to minimize redundancy and preserve model stability, particularly considering instances of low event counts [18,22,25]. Operative time and admission-to-operation interval were incorporated into the regression model as clinically relevant perioperative exposures, given their previously demonstrated associations with postoperative complications [4,9]. Model calibration was evaluated using the Hosmer-Lemeshow goodness-of-fit test. For continuous predictors independently associated with AAE, a baseline-covariate-adjusted threshold analysis was performed by systematically evaluating multiple candidate cut-points across the distribution to identify the inflection value associated with the greatest statistically significant OR. To account for multiplicity arising from this approach, Benjamini-Hochberg correction was applied. Statistical significance was defined as any finding with a terminal p-value <.05.

## Results

### Propensity matching and cohort characteristics

A total of 5808 patients met initial inclusion criteria for the SF cohort, compared with 281 patients in the C1 cohort. Following 1:1 PSM, 281 patients remained in each group and with well-balanced covariates (Table 1). In the matched sample, the SF cohort had a mean age of 50.2±10.6 years, body mass index of 28.1±6.1 kg/m^2^ and was 52.7% male. Corresponding values in the C1 cohort were a mean age of 50.7±13.0 years, body mass index of 27.8±5.6 kg/m^2^, and 52.7% male, respectively.

**Table 1:** Baseline demographics and comorbidities of the SF and C1 multi-level posterior cervical-only fusion and instrumentation cohorts before and after 1:1 propensity matching.

Cohort variable	Before matching			After matching		
	SF cohort	C1 cohort	SMD	SF cohort	C1 cohort	SMD
Patients (N)	5,808	281		281	281	
Male sex	3,408 (58.7%)	148 (52.7%)	**0.122**	148 (52.7%)	148 (52.7%)	<0.001
Age (y)	45.5 (11.4)	50.7 (13.0)	**0.428**	50.2 (10.6)	50.7 (13.0)	0.046
Body mass index (kg/m^2^)	29.8 (6.4)	27.8 (5.6)	**0.339**	28.1 (6.1)	27.8 (5.6)	0.050
Diabetes mellitus	1,305 (22.5%)	51 (18.2%)	**0.107**	53 (18.9%)	51 (18.2%)	0.018
Smoking	1,383 (23.8%)	48 (17.1%)	**0.167**	52 (18.5%)	48 (17.1%)	0.037
Chronic obstructive pulmonary disease	388 (6.7%)	22 (7.8%)	0.044	23 (8.2%)	22 (7.8%)	0.013
Ascites	0 (0%)	0 (0%)	<0.001	0 (0%)	0 (0%)	<0.001
Congestive heart failure	89 (1.5%)	7 (2.5%)	0.071	9 (3.2%)	7 (2.5%)	0.043
Hypertension requiring medication	3,537 (60.9%)	161 (57.3%)	0.073	157 (55.9%)	161 (57.3%)	0.029
Dialysis-dependent renal failure	54 (0.9%)	2 (0.7%)	0.024	1 (0.4%)	2 (0.7%)	0.049
Chronic steroid use	290 (5.0%)	20 (7.1%)	0.089	22 (7.8%)	20 (7.1%)	0.027
ASA classification (≥3)	3,986 (68.6%)	233 (82.9%)	**0.338**	232 (82.6%)	233 (82.9%)	0.009

Continuous variables are reported as mean (SD) and categorical variables as N (%). Bolded standardized mean difference (SMD) values >0.1 indicate meaningful between-group imbalance.

ASA, American Society of Anesthesiologists

Relatively lower-frequency comorbidities, including chronic obstructive pulmonary disease, ascites, congestive heart failure, dialysis-dependent renal failure, and chronic steroid use, were each present in less than 10% of patients in both cohorts. More prevalent comorbidities included diabetes mellitus (18.9% in the SF cohort vs. 18.2% in the C1 cohort), smoking (18.5% vs. 17.1%), and hypertension requiring medication (55.9% vs. 57.3%), with similar distributions between groups, respectively. Notably, ASA classification was binarized to distinguish more severe (ASA ≥3) from less severe (ASA <3) cases, consistent with prior NSQIP-based spine literature, and approximately 82% of patients in each cohort belonged to the more severe classification (Table 1) [26,27].

**Fig. 1:**
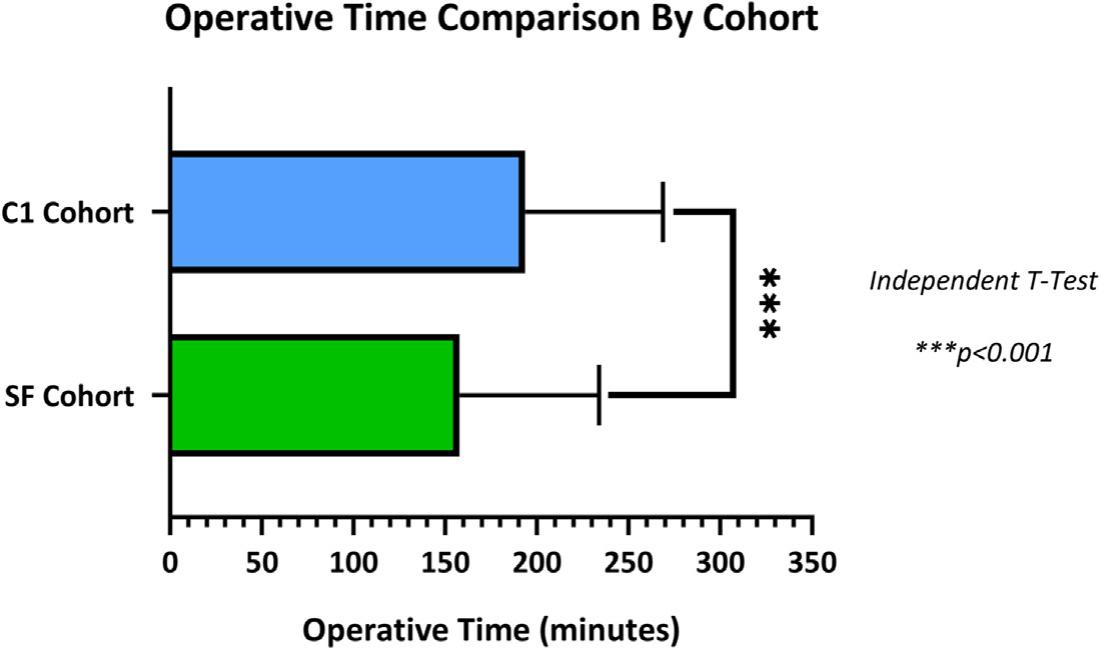
Comparison of mean operative times between the SF and C1 cohorts. Error bars represent standard deviations.

In the SF cohort, patients undergoing multilevel posterior cervical-only fusion and instrumentation had a mean admission-to-operation interval of 0.6±1.8 days, operative time of 157.8±76.1 minutes, and hospital length of stay of 4.5±10.2 days. In comparison, patients in the C1 cohort had corresponding values of 1.5±2.6 days (p<.001), 193.7±74.9 minutes (p<.001; Fig. 1), and 5.3±10.0 days (p=.321). These clinical characteristics, alongside additional demographics, are presented in Table 2.

**Table 2:** Clinical characteristics and additional demographics of patients in the SF and C1 cohorts. SD, standard deviation.

Cohort variable	SF cohort mean (SD) or N (%)	C1 cohort mean (SD) or N (%)	p-value
Patients	281	281	
Race			.085
*White*	213 (75.8%)	231 (82.2%)	
*Black or African American*	37 (13.2%)	24 (8.5%)	
*American Indian or Alaska Native*	3 (1.1%)	1 (0.4%)	
*Asian*	3 (1.1%)	8 (2.9%)	
*Native Hawaiian or Pacific Islander*	1 (0.4%)	2 (0.7%)	
*Unknown/Not reported*	24 (8.5%)	15 (5.3%)	
Inpatient status	275 (97.9%)	274 (97.5%)	.779
Functional status			.277
*Independent*	261 (93.9%)	250 (89.0%)	
*Partially dependent*	17 (6.1%)	27 (9.6%)	
*Totally dependent*	3 (1.1%)	4 (1.4%)	
Admission-to-operation interval (d)	0.6 (1.8)	1.5 (2.6)	**<.001**
Operative time (min)	157.8 (76.1)	193.7 (74.9)	**<.001**
Total length of stay (d)	4.5 (10.2)	5.3 (10.0)	.321

### Comparisons of adverse events

The overall 30-day composite AAE rate was 13.9% in the SF cohort and 18.9% in the C1 cohort (Table 3). Major complications occurred in 11.0% versus 14.6% of patients, respectively, whereas minor adverse events occurred in 6.1% versus 7.1%. Infection rates were low in both groups (4.3% vs. 6.1%, respectively). Bleeding requiring transfusion represented the most common individual complication in each cohort (4.6% in SF vs. 6.1% in C1). Among SF patients requiring ROR, the most frequent causes were wound-related and other musculoskeletal injury (each 28.6%). In the C1 cohort, the leading causes of ROR were implant/mechanical failure and postoperative infection (each 25.0%). Notably, no significant differences were observed between cohorts across all complication-related variables, where compared (Table 3).

**Table 3:** Thirty-day adverse event-related comparisons between the SF and C1 cohorts from 2014 to 2023.

Cohort variable	SF cohort N (%)	C1 cohort N (%)	p-value
Any adverse event	39 (13.9%)	53 (18.9%)	.111
Major adverse event	31 (11.0%)	41 (14.6%)	.207
Minor adverse event	17 (6.1%)	20 (7.1%)	.610
Infections	12 (4.3%)	17 (6.1%)	.340
*Superficial surgical site infection*	0 (0%)	3 (1.1%)	.249
*Deep surgical site infection*	0 (0%)	3 (1.1%)	.249
*Organ/Space surgical site infection*	2 (0.7%)	1 (0.4%)	1.000
*Urinary tract infection*	7 (2.5%)	9 (3.2%)	.612
*Sepsis*	4 (1.4%)	3 (1.1%)	1.000
Wound dehiscence	3 (1.1%)	0 (0%)	.249
Acute kidney injury	0 (0%)	1 (0.4%)	1.000
Pneumonia	7 (2.5%)	8 (2.9%)	.794
Unplanned Intubation	2 (0.7%)	4 (1.4%)	.686
Prolonged ventilator support (>48 h)	3 (1.1%)	4 (1.4%)	1.000
Pulmonary embolism	2 (0.7%)	6 (2.1%)	.286
Cerebrovascular accident/stroke	1 (0.4%)	1 (0.4%)	1.000
Cardiac arrest	1 (0.4%)	2 (0.7%)	1.000
Myocardial infarction	1 (0.4%)	1 (0.4%)	1.000
Deep vein thrombosis	4 (1.4%)	5 (1.8%)	1.000
Bleeding requiring transfusion	13 (4.6%)	17 (6.1%)	.453
Return to operating room (rate)	7 (2.5%)	12 (4.3%)	.243
*Implant/Mechanical failure*	1 (14.3%)	3 (25.0%)	
*Neurological injury*	1 (14.3%)	0 (0%)	
*Cervical fracture*	0 (0%)	1 (8.3%)	
*Postoperative infection*	0 (0%)	3 (25.0%)	
*Wound-related*	2 (28.6%)	0 (0%)	
*Other musculoskeletal injury*	2 (28.6%)	2 (16.7%)	
*Respiratory failure*	0 (0%)	1 (8.3%)	
*Unspecified*	1 (14.3%)	2 (16.7%)	
30-d mortality	2 (0.7%)	4 (1.4%)	.686

### Independent predictors of adverse events

Exploratory multivariable regression illustrated that increasing operative time (per minute, OR=1.009; p<.001) and longer admission-to-operation interval (per day, OR=1.226; p=.016) were independently associated with AAE in the SF cohort (Table 4). No independent predictors were observed in the C1 cohort (Table 5). Hosmer-Lemeshow testing indicated adequate calibration for the SF (χ²=7.237, df=8, p=.511) and C1 (χ²=7.282, df=8, p=.506) models, respectively.

**Table 4:** Multivariable logistic regression identifying independent predictors of AAE in the SF cohort, with odds ratios representing associations with AAE.

SF predictor	Odds ratio	95% confidence interval	p-value
Operative time (per min)	1.009	(1.004–1.013)	**<.001**
Admission-to-operation interval (per d)	1.226	(1.039–1.446)	**.016**
Chronic steroid use	2.469	(0.734–8.309)	.144
Hypertension requiring medication	0.696	(0.307–1.577)	.385
Congestive heart failure	1.366	(0.523–3.567)	.524
Chronic obstructive pulmonary disease	2.803	(0.801–9.812)	.107
Smoking	0.453	(0.143–1.435)	.179
Diabetes mellitus	0.563	(0.188–1.687)	.305
Body mass index (per kg/m^2^)	0.933	(0.862–1.010)	.086
Age (per y)	1.035	(0.993–1.079)	.103
Male sex	0.926	(0.433–1.978)	.842

**Table 5:** Multivariable logistic regression identifying independent predictors of AAE in the C1 cohort, with odds ratios representing associations with AAE.

C1 predictor	Odds ratio	95% confidence interval	p-value
Operative time (per min)	1.003	(0.999–1.007)	.160
Admission-to-operation interval (per d)	1.052	(0.937–1.182)	.392
Chronic steroid use	1.528	(0.512–4.560)	.447
Hypertension requiring medication	1.390	(0.691–2.794)	.356
Congestive heart failure	0.000	(0.000–Undefined)	.999
Chronic obstructive pulmonary disease	1.409	(0.452–4.387)	.555
Smoking	0.879	(0.343–2.249)	.787
Diabetes mellitus	1.150	(0.516–2.564)	.732
Body mass index (per kg/m^2^)	0.970	(0.914–1.030)	.323
Age (per y)	1.022	(0.992–1.052)	.151
Male sex	0.692	(0.367–1.306)	.256

### Risk thresholds for adverse events

**Fig. 2:**
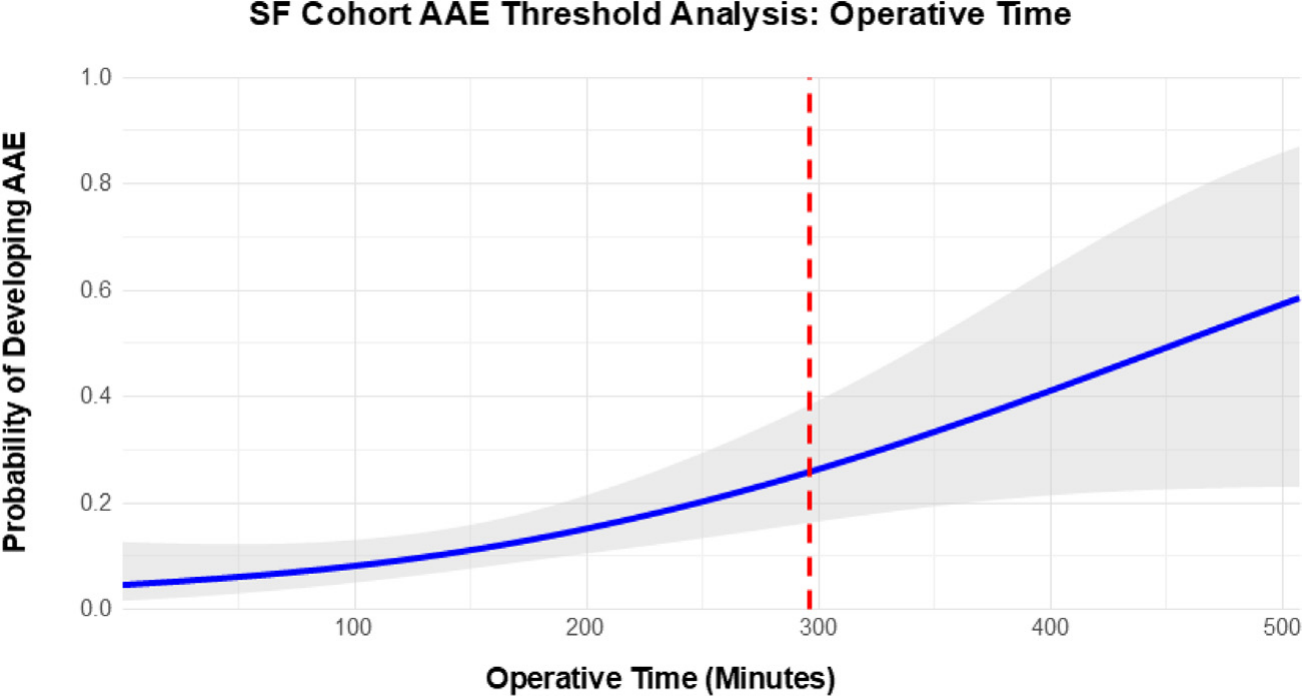
Relationship between operative time and the probability of developing any adverse event (AAE) following multi-level subaxial cervical-only fusion and instrumentation. The dashed red line marks the 296-minute threshold, corresponding to the 95th percentile of the operative time distribution. The blue curve represents the mean predicted probability of AAE across operative times, with gray shading indicating 95% confidence intervals.

Given their independent associations with AAE in the SF cohort, operative time and admission-to-operation interval were further examined using baseline-covariate-adjusted systematic threshold analyses to identify clinically meaningful inflection points. For operative time, a threshold of 296.0 minutes was identified, corresponding approximately to the 95th percentile of the distribution (median=143 minutes; interquartile range [IQR]=106-193). Patients with operative times below this threshold had an AAE rate of 12.0%, compared with 50% among those exceeding it (OR=8.112; 95% CI=[2.519, 26.665]; p<.001; corrected p=.009; Fig. 2). For admission-to-operation interval, a threshold of 0.98 days was identified, approximating the 81st percentile (median=0 days; IQR=0-0). The AAE rate was 10.6% below this threshold versus 27.8% above (OR=3.238; 95% CI=[1.511, 6.820]; p=.002; corrected p=.008).

## Discussion

This study examined differences in 30-day adverse events between multi-level posterior cervical-only fusion and instrumentation constructs extending to C1 versus those confined to the subaxial spine. Findings appeared to demonstrate no clear, significant differences in 30-day AAEs, rates of major and minor complications, infections, or early mortality between cohorts. While prior registry data comparing occipitocervical and atlantoaxial fusion suggested that more proximal construct extension escalates complication risk, our findings indicate that this relationship may not entirely apply to multi-level constructs stopping at C1 versus those confined to the subaxial spine [14]. Though potentially informative, these findings should be interpreted with appropriate caution given important limitations, particularly those related to sample size, statistical robustness, and indication-based constraints.

Interestingly, operative time and admission-to-operation interval were the only independent predictors of AAE in the SF cohort on exploratory multivariable regression. Prolonged operative duration likely reflects case complexity and intraoperative challenges to some extent, including blood loss and tissue manipulation, all of which have been associated with postoperative morbidity in spine surgery [[28], [29], [30], [31]]. Consistent with this, longer operative times have also been linked to increased risk of infection and greater anesthetic exposure, suggesting that operative efficiency may be a potentially modifiable contributor to early postoperative adverse outcomes [28,[32], [33], [34]].

Similarly, prolonged admission-to-operation interval may also serve as a proxy for acute presentation severity or medical instability requiring preoperative optimization. However, variables reflecting acute physiological insult, including sepsis, recent transfusion, and ventilator dependence, were controlled for in the selection process to minimize confounding from preoperative instability, suggesting that additional factors not captured by the database may have contributed to this finding. In support of these results, prior work has demonstrated that operative delays in fusion populations have been shown to lead to worse postoperative outcomes [35,36]. Though notably, neither operative time nor admission-to-operation interval reached significance in the C1 cohort, which may reflect limited power, differences in operative indications, or distinct perioperative risk drivers. Nevertheless, the present study’s findings warrant further investigation in more robust, granular cohorts with greater indication-specificity to corroborate and expand upon inferences made.

To further characterize the aforementioned temporal predictors, threshold analyses were subsequently performed. This identified clinically relevant inflection points at 296 minutes of operative time and an admission-to-operation interval of approximately one day in SF patients, above which AAE rates increased substantially. Under typical circumstances, the majority of patients fall below these thresholds, as they correspond roughly to the 95th and 81st percentiles of their respective distributions. However, when these thresholds are exceeded, the perioperative consequences appear to increase significantly. While prospective validation is necessary to apply these thresholds, they offer a starting point for identifying SF patients at elevated risk and may help guide preoperative optimization. Notably, the admission interval threshold near one day raises the possibility that delayed intervention contributes to early morbidity in the SF population; however, it is important to recognize that causal inference cannot be established in this manner, especially given the inability to fully account for surgical indication within the NSQIP framework.

Beyond temporal predictors, evaluation of individual complication domains revealed that infection rates were low and plausibly statistically comparable across both cohorts (4.3% in SF vs. 6.1% in C1 cases). This finding is notable given the longer operative times observed in the C1 cohort, as procedure duration has been consistently associated with increased infection risk in spine surgery [32,33]. Despite longer operative times in the C1 cohort, infection rates were not significantly different between groups, cautiously suggesting that atlantoaxial fusion and instrumentation itself may not confer an increased risk of postoperative infection. This may reflect the influence of prophylactic antibiotics, wound management protocols, or institutional practices that attenuate infection risk even in longer, complex operations [[37], [38], [39], [40]]. Although remaining vigilant about infection prevention is important, the present data does not clearly support the notion that constructs extending through C1 represent independent infectious risks to consider during operative planning; therefore, further corroborative study remains necessary.

Taken together, these findings seemingly suggest that in the context of multi-level posterior cervical-only fusion and instrumentation, proximal construct extent does not appear to clearly drive short-term adverse events. Instead, temporal factors such as operative time and preoperative admission interval appear to be more potentially actionable determinants of early morbidity, particularly within the SF cohort. These findings carefully underscore the importance of efficiency in cervical spine surgery and can help to reassure surgeons that a construct through the atlantoaxial joint does not necessarily entail higher short-term risk. Future, larger studies with longer follow-up, indication-specific analyses, and surgeon- and hospital-level variables are imperative for expanding upon such findings and verifying reported results.

### Limitations

Though relatively novel and exploratory, the present study’s findings should be interpreted while considering several important limitations. First, NSQIP does not record outcomes or other variables beyond thirty days, making it difficult to extrapolate longer-term findings that are relevant to cervical fusions such as revision surgery rates, implant complications, and changes in patient-reported outcome measures (PROMs). In addition, NSQIP lacks granularity about specific construct characteristics such as the exact levels involved, implant types, radiographic parameters, operative indication severity, and surgeon- or facility-level factors that could have influenced the results of this study.

Regarding statistical power, although NSQIP provides a large, multi-institutional registry well-suited for evaluating low-frequency adverse events, and a decade’s worth of data was pooled with appropriate selection criteria to isolate and efficaciously match the much smaller C1 cohort, the resulting sample size for this subgroup and ultimately the larger analysis was relatively small. As such, the present study may be underpowered to detect true meaningful differences despite the use of exact statistical methods where necessary, and nonsignificant findings should therefore not be interpreted as definitive evidence of equivalence or true convergence between groups. Rather, these findings should be interpreted as hypothesis-generating and within the context of such limitations.

In a similar vein, data-modelling-related considerations also invite interpretative caution. For example, the multivariable regression may have been susceptible to overfitting given the relatively low number of events in relation to the number of covariates included, increasing the risk for instability and sparse-data bias. While covariates were preselected in accordance with established NSQIP-leveraging literature, and model assessment using Hosmer-Lemeshow testing did not suggest a lack of fit, these approaches do not fully mitigate such risks, and thus regression estimates should also be interpreted carefully and as exploratory ventures. This is especially relevant when considering the lack of significant predictors of AAEs in the C1 cohort’s regression, which may be a reflection of insufficient power rather than a natural absence of associations, and thus warrants further investigation in larger cohorts, when feasible.

Furthermore, as with any large registry study, this analysis is also limited by variables not reliably captured within the database infrastructure, which is particularly important in the context of comorbidities and underlying etiologies for operation. One such comorbidity-based example is rheumatoid arthritis, which is a well-established contributor to atlantoaxial instability and potential indication for construct extension to C1 [41]. Irrespective of its clinical relevance, however, this condition is not dependably or granularly captured within the current iterations of the NSQIP database and therefore could not be controlled for or analyzed. Accordingly, rheumatoid arthritis, along with other inflammatory conditions, potential diagnoses/indications for posterior cervical fusion and instrumentation, and other unmeasured variables may have influenced observed outcomes and should be taken into account when appraising findings. Conversely, these limitations also highlight opportunities for complementary investigations in settings where reliable ICD-10-based diagnoses and indication capture, such as for trauma or deformity cases, are more robust and can be effectively integrated into analyses. Another limitation inherent to large administrative databases such as NSQIP is that comorbidities are recorded as dichotomous variables, which limits insight into disease severity, duration, or treatment history. While this is a common constraint here, it may introduce residual confounding that cannot be fully accounted for.

Despite these limitations however, and to the authors’ best knowledge, this study represents the largest contemporary analysis comparing short-term outcomes between multi-level posterior cervical-only fusion and instrumentation constructs extending to C1 and those confined to the subaxial cervical spine. As such, these early findings provide an exploratory foundation for understanding perioperative risk that future, longer-term, and prospective studies can build upon.

## Conclusion

In this national NSQIP-based analysis, extending a multi-level posterior cervical-only fusion and instrumentation construct to C1 did not appear to be clearly associated with an increase in 30-day adverse events when compared to constructs confined to the subaxial cervical spine. Notably within the SF cohort, operative time beyond 296 minutes and preoperative admission beyond approximately 1 day were associated with substantially higher complication rates, highlighting perioperative timing as a potentially modifiable risk factor, when setting-appropriate. These same exploratory temporal factors did not reach significance within the C1 cohort despite significantly longer operative durations, potentially suggesting that short-term risk in atlantoaxial constructs may not be driven by the same registry-specific perioperative variables captured in such a manner, although insufficient power may have also contributed to these findings. Given the strict 30-day window of NSQIP, among the many other statistical- and database-related limitations encountered, cautious interpretation is warranted. Larger, longer-term, and indication-specific investigations are needed to corroborate these results and confirm whether differences in outcomes emerge beyond the early postoperative period. Even so, the current analysis may help to offer nuanced insights into surgical decision-making, perioperative risk stratification, and patient counseling when considering proximal extension to C1.

## Funding

No funding was received for this study.

## Ethical approval

The use of fully deidentified data qualified this study as nonhuman subjects research and therefore institutional review board approval, informed consent, or other ethical considerations were not required.

## Illustrations

The figures provided in this manuscript are intended for publication in color.

## CRediT authorship contribution statement

**Carla Guedikian:** Conceptualization, Formal analysis, Investigation, Visualization, Writing – original draft, Writing – review & editing. **Shahabeddin Yazdanpanah:** Conceptualization, Formal analysis, Data curation, Investigation, Methodology, Software, Visualization, Writing – original draft, Writing – review & editing. **Hana-Joy E. Hanks:** Formal analysis, Validation, Writing – original draft, Writing – review & editing. **Sultan Baz:** Formal analysis, Validation, Writing – original draft, Writing – review & editing. **Yu-Po Lee:** Conceptualization, Visualization, Project administration, Resources, Writing – review & editing. **Emily S. Mills:** Conceptualization, Visualization, Project administration, Resources, Writing – review & editing. **Nitin N. Bhatia:** Conceptualization, Visualization, Project administration, Resources, Writing – review & editing. **Don Y. Park:** Conceptualization, Visualization, Project administration, Resources, Writing – review & editing. **Sohaib Z. Hashmi:** Conceptualization, Visualization, Project administration, Resources, Writing – review & editing. **Hao-Hua Wu:** Conceptualization, Visualization, Project administration, Resources, Writing – review & editing.

## Declaration of competing interests

The authors declare that they have no known competing financial interests or personal relationships related to this study that could have appeared to influence the work reported in this paper.
